# Proteomic Profiling Reveals Mitochondrial Dysregulation in Rapidly Progressive Alzheimer’s: Role of DLDH in Amyloid Beta Aggregation

**DOI:** 10.1007/s12035-025-05327-0

**Published:** 2025-11-19

**Authors:** Saima Zafar, Aneeqa Noor, Neelam Younas, Mohsin Shafiq, Kathrin Dittmar, Oleksandr Yagensky, Matthias Schmitz, Isidre Ferrer, Peter Hermann, Inga Zerr

**Affiliations:** 1 Department of Neurology, Clinical Dementia Centerand, DZNE, Georg-August University, University Medical Center Goettingen (UMG), Robert-Koch-Str. 40, Goettingen, 37075 Germany; 2 Biomedical Engineering and Sciences Department, School of Mechanical and Manufacturing Engineering (SMME), National University of Sciences and Technology (NUST), Bolan Road, Islamabad, H-12, 44000 Pakistan; 3 Institute of Neuropathology, University Medical Center Hamburg-Eppendorf, Hamburg, Germany; 4 Department of Neurobiology, Max Planck Institute for Biophysical Chemistry, Goettingen, Germany; 5 Institute of Neuropathology, IDIBELL-University Hospital Bellvitge, University of Barcelona, L’Hospitalet de Llobregat, Barcelona, Spain

**Keywords:** Alzheimer’s disease (AD), Rapidly progressive Alzheimer’s disease (rpAD), Proteomics, Metabolism, DLDH, Post-translational modification, Metabolic networks

## Abstract

Alzheimer’s disease (AD) is presented as multiple clinical variants depending upon the rate of progression and familial background; however, the exact molecular mechanisms associated with these subtypes and their treatments are yet to be understood. The current study is based on a global proteome analysis of brain samples from patients (*n* = 38) with rapidly progressive AD (rpAD—survival time < 3 years), sporadic AD (spAD—survival time of 8–10 years), and healthy controls. Proteome analysis revealed a differential regulation of 79 proteins and highlighted the dysregulation of mitochondrial machinery and glucose metabolism in rpAD. Dihydrolipoamide dehydrogenase (DLDH), a mitochondrial oxidoreductase, showed a significant reduction and delocalization in rpAD. In vitro analysis revealed a potential role of DLDH in the aggregation of amyloid beta. Rapid progression in AD may be influenced by the energy homeostasis and redox dysfunction linked with the DLDH.

**Supplementary Information:**

The online version contains supplementary material available at 10.1007/s12035-025-05327-0.

## Introduction

Alzheimer’s disease (AD) is a degenerative neurological disorder affecting millions of people worldwide. It is characterized by progressive cognitive decline and memory loss [1]. AD exists as multiple clinical variants, distinguished based on age at onset, duration of disease, and familial history. Among these clinical variants, rapidly progressive AD (rpAD) has been most recently discovered. It presents a faster decline in cognition (> 6 Mini-Mental State Examination points per year) in contrast to sporadic AD (spAD) (3–6 Mini-Mental State Examination points per year), resulting in death within 3 years (weeks in some cases) of the disease onset. However, the underlying mechanisms are similar, and no associated genetic alterations have been reported to date [2, 3]. Several attempts to identify the discriminating features have been conducted so far, but a detailed proteomic evaluation of metabolic pathways is lagging to date [4–7].

Proteomic alterations in spAD disturb an array of pathways leading to the formation of neurofibrillary tangles, accumulation of amyloid beta (Aβ) plaques, aberrations in membrane permeability, synaptotoxicity, mitochondrial dysfunction, and oxidative stress [8, 9]. Oxidative stress occurs due to the imbalance between reactive oxygen species (ROS) generation and their scavenging mechanisms, attributed to the changes in peroxidases and antioxidant enzymes [10].

Dihydrolipoyl dehydrogenase (DLDH) is a flavoenzyme that plays a vital role in the maintenance of cellular redox homeostasis by transferring electrons in the mitochondrial respiratory chain and reducing NAD + to NADH [11]. It is a key component of three dehydrogenase complexes in mitochondria, namely, the pyruvate dehydrogenase complex, α-keto glutarate dehydrogenase complex, and branched chain amino acid dehydrogenase complex. Its primary role in all three pathways is the conversion of dihydrolipoamide to lipoamide, which further contributes toward glucose metabolism [12]. It contributes toward oxidative stress through the oxidation of flavin adenine dinucleotide and the production of ROS [13].

It is widely believed that the primary function of DLDH is pro-oxidant in nature, accomplished by reducing O_2_ to a superoxide radical or ferric to ferrous iron, which then catalyzes the production of hydroxyl radical through Fenton chemistry [14]. Nevertheless, the diaphorase activity of DLDH, which is capable of scavenging nitric oxide and reducing ubiquinone to ubiquinol, suggests a possible antioxidant role [15–17]. The oligomeric state of DLDH is pH-dependent, transitioning from dimeric to monomeric or tetrameric forms in the mitochondrial matrix. Changes in the oligomeric state have been shown to correlate with a shift from DDL activity (present only in dimeric and tetrameric forms) to diaphorase activity (present in all forms of the protein) [18, 19]. DLDH therefore represents a highly versatile oxidoreductase that plays multiple critical roles in energy metabolism and redox balance [18, 20–22].

Recent studies have suggested that DLDH may play a crucial role in the pathogenesis of AD. Studies have shown that impaired DLDH activity contributes to oxidative damage in the brain, leading to neuronal toxicity and ultimately the development of AD [23]. In contrast, the enhanced activity of DLDH can protect the neurons from oxidative stress injury and improve cognitive function [24]. Furthermore, studies have indicated that the downregulation of the genes encoding DLDH could make the brain more susceptible to oxidative damage and accelerate AD pathogenesis [25].

Owing to the role of DLDH in the pathogenesis of AD, we hypothesize that the evaluation of redox metabolism through in-depth proteomic analysis can reveal aberrations that can account for rapid progression attributed to rpAD. The current study identified altered proteome signatures and identified risk factors by applying a differential proteomics approach in concert with molecular and morphological techniques to elucidate the changes and risk factors involved in the slow and rapid progression of AD pathology.

## Materials and Methods

### Collection of Brain Samples

AD and rpAD brain tissues (frontal cortex) were collected from the Institute of Neuropathology Brain Bank, Barcelona (HUB-ICO-IDIBELL Biobank) in accordance with Spanish legislation (Ley de la Investigación Biomédica 2013 and Real Decreto Biobancos, 2014).

The neuropathological assessment of spAD (*n* = 13; mean age = 75.9 ± 9.4 years), rpAD (*n* = 15; mean age = 78.5 ± 5.9 years), and age-matched non-demented controls (*n* = 10; mean age = 70.9 ± 7.1 years) was performed as described previously [26, 27]. Patients with a family history of dementia and those with comorbidities were excluded. ApoE genotyping was performed using commercially available kits (DNA STRIP, Bruker, Germany) as per the manufacturer’s instructions. The clinical data for all the samples used in this study have been summarized in Table 1.

**Table 1:** Summary of samples used in the current study. The gender, age (years), disease duration (years), and Braak stages of rpAD, spAD, CJD, and control cases utilized in this study

Nr	Patients ID	Gender	Age	Disease duration (years)	Braak stages (AD)
1	rpAD1	Male	70	< 4	VI C
2	rpAD2	Male	76	< 4	VI C
3	rpAD3	Female	76	< 4	VI C
4	rpAD4	Female	77	< 4	IV/A
5	rpAD5	Male	78	< 4	V/C
6	rpAD6	Female	79	< 4	V
7	rpAD7	Female	81	< 4	III/B
8	rpAD8	Male	83	< 4	VI C
9	rpAD9	Male	83	< 4	V/C
10	rpAD10	Female	85	< 4	V
11	rpAD11	Female	85	< 4	IV
12	rpAD12	Male	65	< 4	–
13	rpAD13	Female	86	< 4	V
14	rpAD14	Female	75	< 4	III
15	rpAD15			< 4	
16	spAD1	Female	56	> 4	V/C
17	spAD2	Male	64	> 4	II/A
18	spAD3	Female	67	> 4	III/C
19	spAD4	Male	69	> 4	III/0
20	spAD5	Female	71	> 4	III/0
21	spAD6	Female	72	> 4	V/C
22	spAD7	Female	75	> 4	V/C
23	spAD8	Male	78	> 4	V/C
24	spAD9	Female	82	> 4	VI/B
25	spAD10	Male	83	> 4	III/0
26	spAD11	Male	87	> 4	V/C
27	spAD12	Female	90	> 4	IV/A
28	spAD13	Male	93	> 4	V/C
29	Control 1	Male	69	–	II/A
30	Control 2	Male	68	–	I/0
31	Control 3	Male	64	–	I/0
32	Control 4	Male	67	–	I/0
33	Control 5	Male	74	–	II/A
34	Control 6	Male	86	–	II/A
35	Control 7	Female	73	–	I/0
36	Control 8	Male	70	–	I/A
37	Control 9	Male	61	–	I
38	Control 10	Male	77	–	I/A

### Proteomic Evaluation

#### Protein Extraction

Protein extraction was performed by the homogenization of brain tissues (10% w/v) in urea-thiourea lysis buffer (7 M urea, 2 M thiourea, 4% CHAPS, 1% DTT, protease, and phosphatase inhibitors). Alternatively, cell pellets were washed and homogenized in the lysis buffer. The homogenate was incubated at 4 °C overnight. The samples were centrifuged, and the supernatant was utilized for analysis after quantification using the Bradford assay (Bio-Rad, USA).

#### Two-Dimensional Electrophoresis (2DE)

Two-dimensional gel electrophoresis was employed to identify differentially altered proteins. Samples were loaded onto pH 3–10, 17 cm, non-linear IPG strips (Bio-Rad, Germany), followed by second-dimension resolution on 17 cm polyacrylamide gels. Silver staining was performed as described previously [28]. Stained gels were imaged using Gel CanoScan 8400 F (Canon, Japan). Delta 2D software version 3.6 (Decodon GmbH, Greifswald, Germany) was used, with differentially expressed spots defined as those showing a ≥ 1.5-fold change and *p* < 0.05 (unpaired Student’s *t*-test) for densitometric analysis.

#### Identification of Differentially Expressed Proteins

The relative intensities of individual spots were normalized to the sum of all spot intensities on the selected gel. Targets with a fold change of ≥ 1.5 and *p* ≤ 0.05 were selected and excised. In-gel protein digestion and tandem mass spectrometric analysis (MS/MS) were performed as described previously [29]. Protein identification was accepted if at least two peptides were sequenced from each protein spot. The results were obtained from four independent experiments.

### Immunoblot Analysis

An immunoblot assay was used for semi-quantitative analysis of selected targets. Proteins were transferred to 0.20 µm PVDF membranes under semi-dry conditions (1 mA/cm^2^, 45 min), washed, blocked (1 h), and incubated in primary antibodies anti-PrP mAb (SAF70, 1:1000, #A03206), anti-amyloid beta A4 protein mAb (1:1000), anti-PrP 3F4 mAb (1:1000), human dihydrolipoamide dehydrogenase/DLDH (1:1000), anti-pyruvate dehydrogenase (1:1000), anti-CALHM6 (1:800), anti-beta actin (1:800), anti-Tau mouse monoclonal (TAU5) (Abcam cat.no. ab80579, RRID: AB_1603723) (0.5 μg/mL), anti-phospho Tau mAb (T231, 1:1000, #ab226492), anti-TIA1 (sc-1751), anti-syntaxin 6 mAb (1:1000, #60059–1), and anti-GAPDH antibody (1:1000, #G8795) overnight at 4 °C. The blots were washed and incubated in a secondary antibody (1 h), followed by signal detection through enhanced chemiluminescence solution and ChemiDoc™ Imaging System (Bio-Rad, Germany).

### Immunofluorescence Confocal Microscopy

Tissue sections (frozen and paraffin-embedded) were stained, and confocal laser scanning microscopy was performed as described previously [30, 31]. Imaging was performed using an LSM510 laser-scanning microscope (Zeiss, Göttingen, Germany; 543 and 633 nm helium–neon and 488 nm argon excitation wavelengths). ImageJ (WCIF plugin) and FIJI (coloc 2) software were used for the individual evaluation of images. Correlations between two targets were performed through threshold Manders’ overlap coefficients (tM1 and tM2) and Pearson’s linear correlation coefficient (rP) [32].

### In Vitro Aggregation Assays

Synthetic amyloid-β (Aβ) was prepared in various aggregation states as described previously [33]. DLD peptide (17 µg) was added per 100 mM of Aβ peptide, and the mixture was incubated for 24 h. A 20 µM ThT dye was added to the mixture, and the absorbance was measured using FLUOstar Omega plate reader (BMG LabTech, Germany).

### Electron Microscopy

Negative staining electron microscopy was performed for structural analysis. Products from in vitro aggregation assays were loaded onto mesh copper grids and stained with uranyl acetate replacement stain (Electron Microscopy Sciences, USA). The resultant aggregates were imaged using a Joel 1011 Electron Microscope (Joel, Germany).

### Generation of Animal Models

All animal experiments were performed in accordance with section 4 of the Animal Welfare Law of the Federal Republic of Germany (section 4 of TierSchG, Tierschutzgesetz der Bundesrepublik Deutschland). Genetic animal models of AD were generated and maintained as described previously [34, 35]. Brain homogenate (20 μL of 10% w/v sample homogenized in PBS) was injected into the thalamus of 10-month-old mice. Both inoculated and non-inoculated animals (*n* = 48) were sacrificed 3-, 6-, 9-, and 12-month post-inoculation via decapitation, and cortical samples were obtained.

### Immunoprecipitation

The identification of DLD interactors was performed using immunoprecipitation. For this assay, brain samples were homogenized in tris lysis buffer (50 mM Tris–Cl [pH 8.0], 0.5% CHAPS, 1% Triton X-100, 1 mM DTT, protease, and phosphatase inhibitors). An 8 µL DLD antibody (Abcam, UK) was coated on magnetic beads (1.5 mg/0.5 mg of protein sample; Dynabeads; Invitrogen, USA) after they were given two washes with 0.3% CHAPS. A 500 µg protein was added to coated beads, and the mixture was incubated overnight. Proteins were eluted in rehydration buffer (8.3 M urea, 0.5% CHAPS, and 20 mM DTT) and run on gels to a length of 1 cm. Bands were excised, and in-gel digestion was performed. Analysis of the eluates was performed using the Top10 method in the data-dependent acquisition mode through a Q Exactive hybrid quadrupole/Orbitrap MS system (paired with Excalibur software). Tandem mass spectra were obtained using Raw2MSM software. Mascot was instructed to search the SwissProt *Homo sapiens* reference proteome (revision 10.2018) with a mass tolerance of 10 ppm for precursors and 0.05 Da for fragments, and the Scaffold software was used for the analysis of spectra. Protein identification was accepted at a confidence threshold greater than 95.0% for peptide identifications and a confidence threshold of 99.0%, paired with a minimum of two identified peptides for protein identification.

### Statistical Analysis

All reported results have been obtained from at least four sets of independent experiments. Statistical analysis and data visualization were performed using PRISM and RStudio. One-way ANOVA followed by Tukey’s post hoc test or unpaired Student’s *t*-test was used to determine *p*-values, and *p* ≤ 0.05 was considered significant. The findings have been presented as mean ± standard deviation (SD) unless stated otherwise.

## Results

### Allele and Genotype Frequency of ApoE in rpAD and spAD Cases

**Fig. 1:**
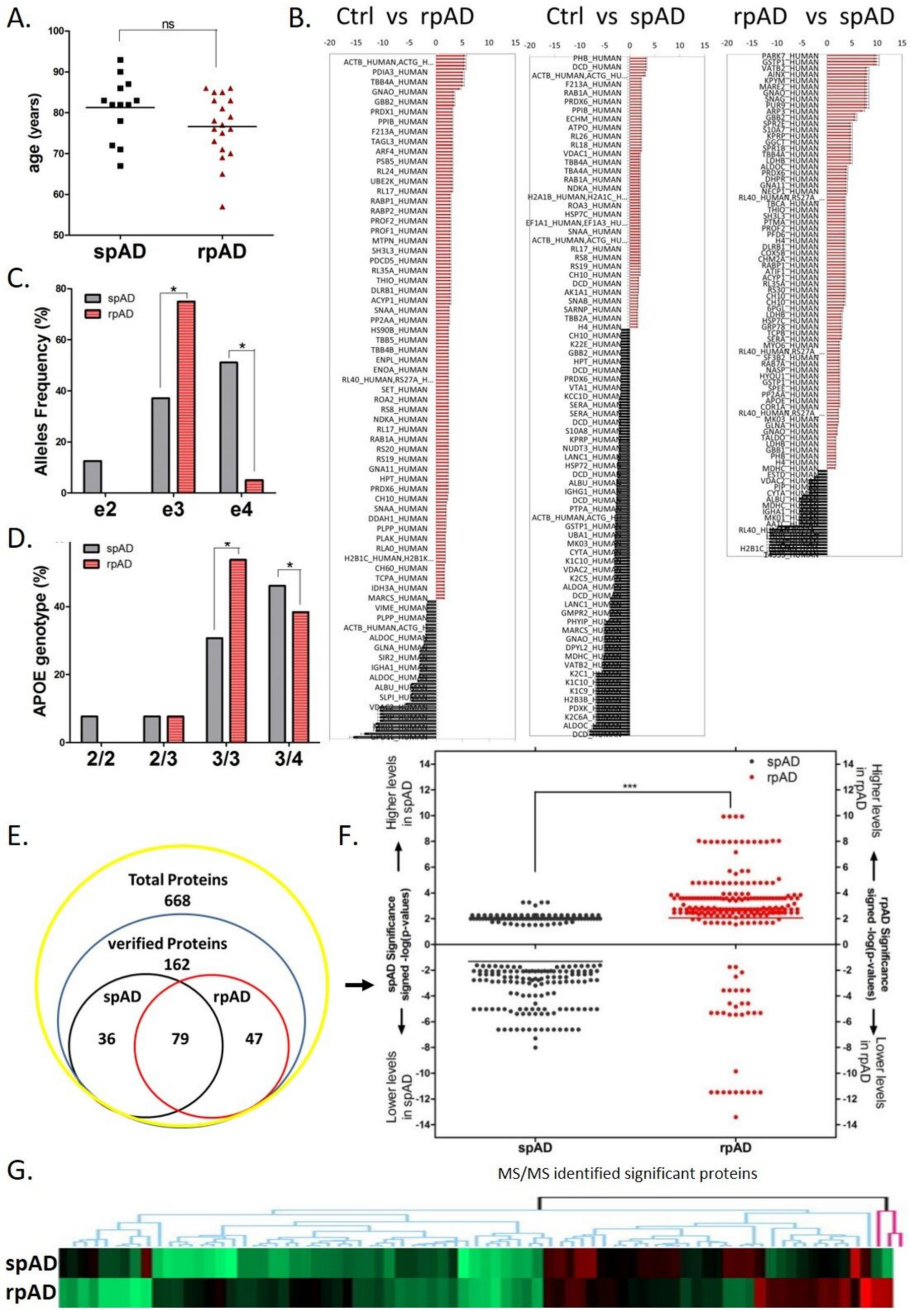
Genomic and proteomic analysis of control, spAD, and rpAD cases. The figure depicts the differences in **A** age at onset, **B** proteomic expression, **C** APOE allele frequency, and **D** APOE genotype among spAD (grey) and rpAD (red) cases. **E** spAD and rpAD presented differential expression in 36 and 47 proteins, respectively. **F**, **G** The relative expression of these targets in the two variants of AD is also depicted. “*” represents *p* < 0.05, “***” represents *p* < 0.001, ns = non-significant

In total, 28 individuals from spAD and rpAD were recruited in our study, with an almost equal male-to-female ratio, and the proportions of APOE genotypes from the representative are listed in Supplementary Table 1. The proportions of APOE ε2/3 and ε3/4 were 7.1% and 64.2%, respectively, in spAD cases. However, in rpAD cases, the proportions of APOE ε3/3 and ε3/4 were 66.6% and 33.3%, respectively. The proportion of homozygous ε2 and ε3 was 7.1% and 21.4%, respectively, only in spAD cases. Furthermore, spAD showed APOE ε2, ε3, and ε4 allele frequencies around 11.5%, 57.6%, and 30.7%, and in rpAD, ε3 showed the highest frequency of 83% and ε4 showed 16.6% (Fig. 1A, C, D).

### Quantitative Proteomic Profiling of rpAD and spAD Cases

**Fig. 2:**
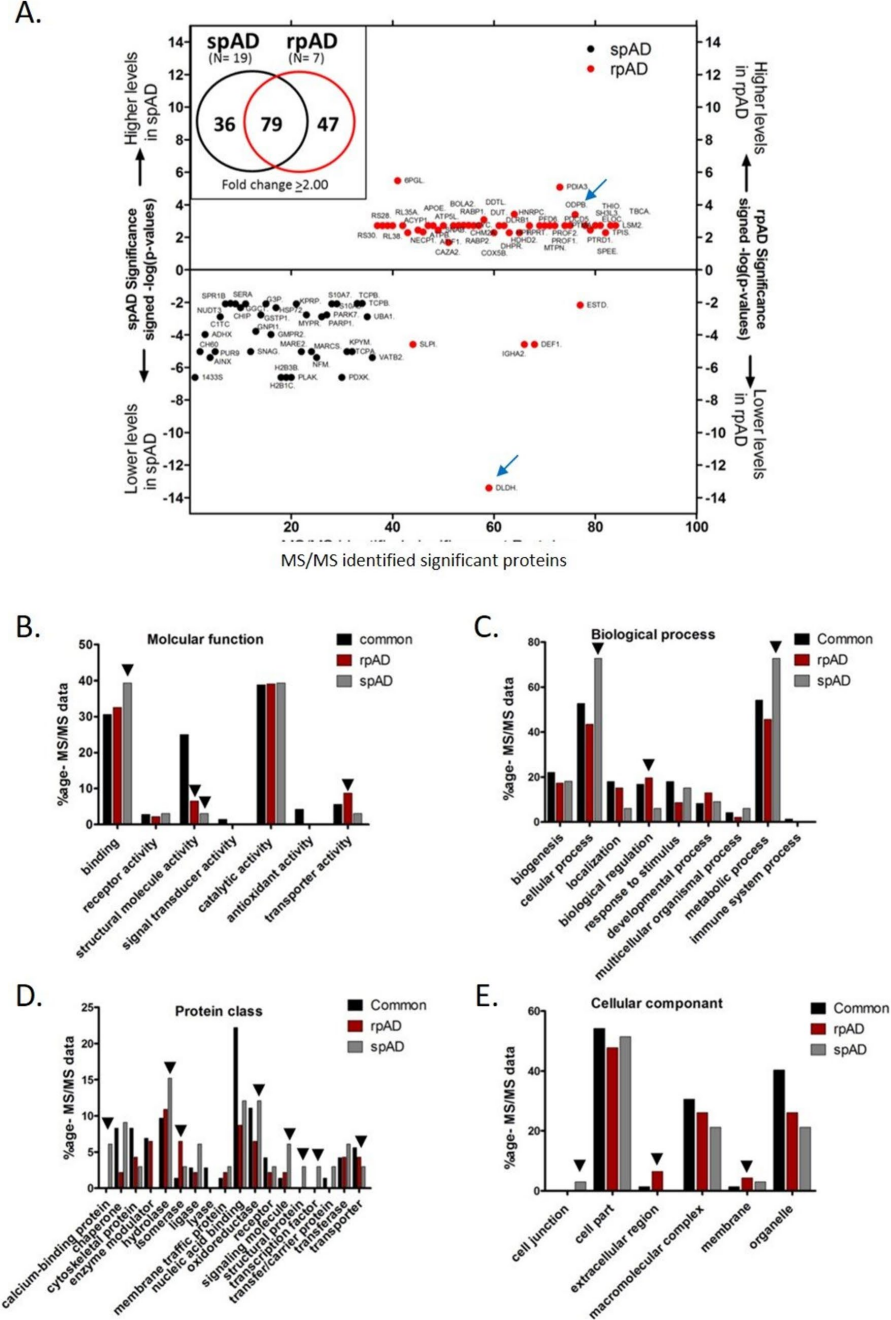
Functional characterization of differentially regulated proteins in control, spAD, and rpAD cases. **A** The relative expression of proteins with significant expression variations and their categorization based on **B** molecular function, **C** biological process, **D** protein class, and **E** cellular location

Proteomic evaluations of the frontal cortex tissues from rpAD, spAD, and control brains were conducted to identify the modulators of progression rate in the clinical variants of AD. The gel-based affinity enrichment and high-resolution label-free Q-TOF LC–MS/MS analysis (Supplementary Fig. 1) revealed differential regulation in rpAD and spAD versus control brain samples (Fig. 1B), and we found a total of 668 differentially regulated proteins (Fig. 1E). We used conservative approaches of selecting only the consensus results of four normalization methods and found 79 common proteins in rpAD and spAD cases that showed significantly altered expression levels (*p*-values < 0.05, corrected for multiplicity of testing; Supplementary Table 2) in comparison to controls. However, 36 and 47 proteins showed specific regulatory levels in spAD and rpAD subjects, respectively (Fig. 1E; Supplementary Tables 3 and 4). Additionally, among the 79 common proteins in rpAD and spAD, rpAD showed expressional changes (either upregulated or downregulated) in most of the proteins in comparison to spAD (Fig. 1F, G). Interestingly, the levels of unique sets of 47 and 36 proteins, respectively, altered in rpAD and spAD showed significantly higher protein levels in rpAD and low levels in spAD brain tissue (Fig. 2A).

Altogether, the categorical analysis related to molecular function showed significantly unique altered levels of proteins involved in structural molecular activity, i.e., 6.5% in rpAD and only 3.0% unique in spAD (Fig. 2B). The categorial analysis of the biological process showed 15.2% unique proteins involved in localization and 13.0% in the developmental process (Fig. 2C). Interestingly, protein class categorical analysis showed significantly altered unique proteins involved in calcium-related function in spAD (Fig. 2D) and significantly altered unique proteins involved in enzyme modulator in rpAD (Fig. 2D). However, the cellular component categorical analysis showed significantly altered unique proteins involved in cell junction in spAD (Fig. 2E) and significantly altered unique proteins involved in the extracellular region and membrane proteins uniquely modified in rpAD (Fig. 2E).

### Differential Regulation of Metabolic Proteins in rpAD

**Fig. 3:**
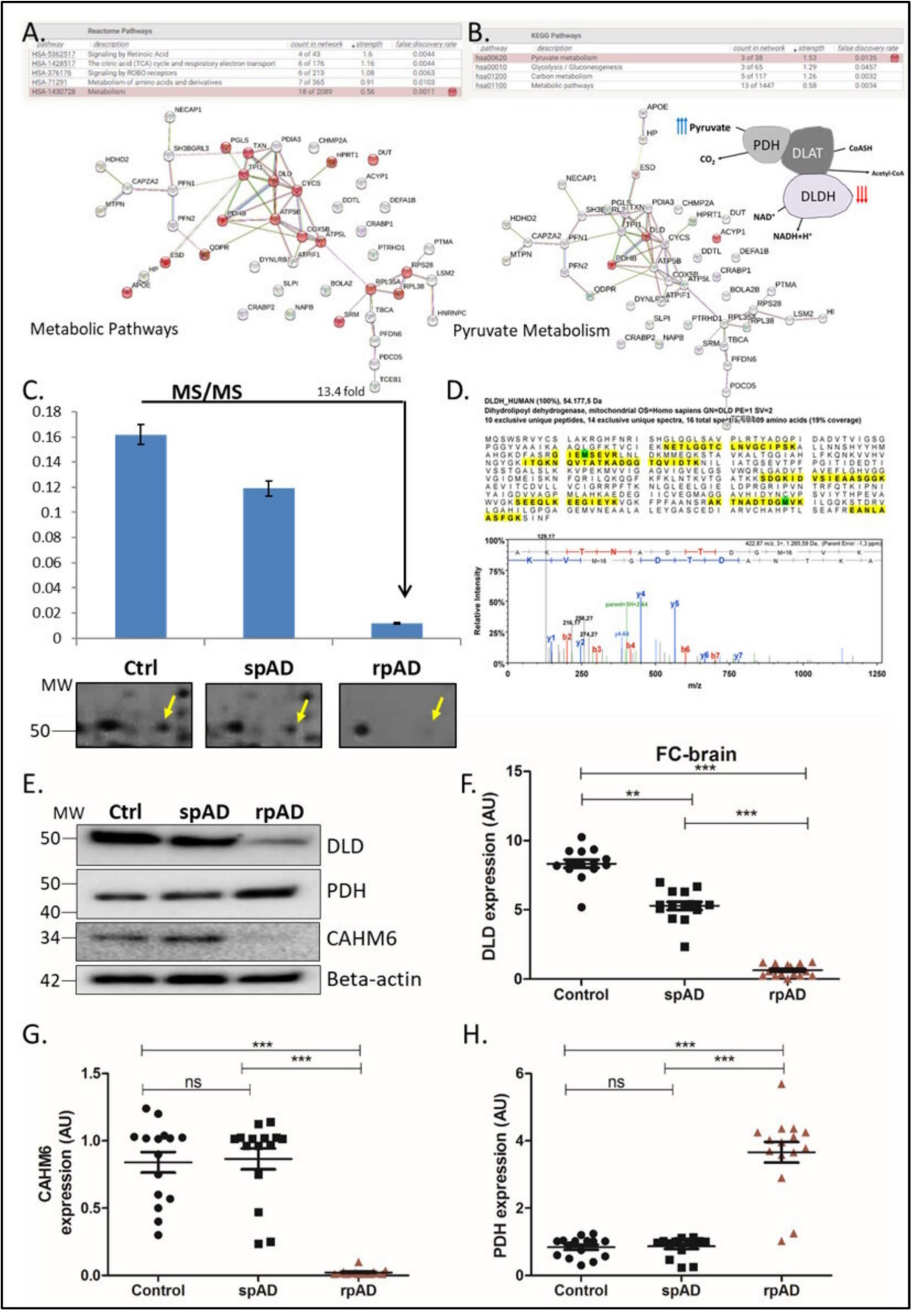
Differential expression of metabolic proteins, especially DLDH, in rpAD cases. Functional characterization and network analysis revealed alterations in **A** metabolic pathways, including **B** pyruvate metabolism. **C** The expression of DLDH, as detected by mass spectrometry and illustrated by the corresponding DLDH spot in the 2DE image (**D**), was significantly reduced in rpAD. Western blot analysis (**E**) also depicted expression variations in DLDH (**F**), CAHM6 (**G**), and PDH (**H**). Data is presented as mean ± SEM. “**” represents *p* < 0.01, “***” represents *p* < 0.001, ns = non-significant

The MS/MS data highlighted the differential regulation of proteins involved in metabolic pathways (Fig. 3A), including pyruvate metabolism (Fig. 3B). We focused our efforts on investigating the proteins involved in mitochondrial machinery and glucose metabolism leading to disrupted ATP energy production because the rpAD samples showed significantly altered levels of proteins, i.e., deoxyuridine 5′-triphosphate nucleotidohydrolase (3.0-fold change), cytochrome c oxidase (2.7-fold change), dihydrolipoyl dehydrogenase (DLDH; 13.4-fold change; Fig. 3C), dihydropteridine reductase (2.2-fold change), haloacid dehalogenase–like hydrolase domain–containing protein 2 (2.7–fold change), and hypoxanthine–guanine phosphoribosyltransferase (2.27-fold change), when compared with spAD cases and control brain tissues (Supplementary Table 4). DLDH, which is a key mitochondrial oxidoreductase, showed the highest significant altered levels in rpAD brain samples and was thereby selected for further evaluation.

These expression differences in DLDH were further validated by Western blot analysis. The reduction in the expression of DLDH was accompanied by an upregulation in the expression of its interactor PDH, indicating a significant alteration in pyruvate metabolism in AD cases depicting rapid progression (Fig. 3E–H). Interestingly, we also found a significant downregulation in calcium homeostasis modulator protein 6 in rpAD brain samples. The 2D gel analysis in Fig. 3C captures specific isoforms of DLD that are reduced in rpAD, whereas the Western blot reflects overall protein expression levels. Differences between the 2D gel (Fig. 3C) and Western blot results (Fig. 3E) likely arise from the distinct methodologies: 2D gel electrophoresis separates proteins based on both isoelectric point and molecular weight, highlighting isoforms or post-translational modifications, while Western blotting detects total protein via antibody specificity.

### Colocalization of DLDH with Tau and Aβ in spAD and rpAD Brains

**Fig. 4:**
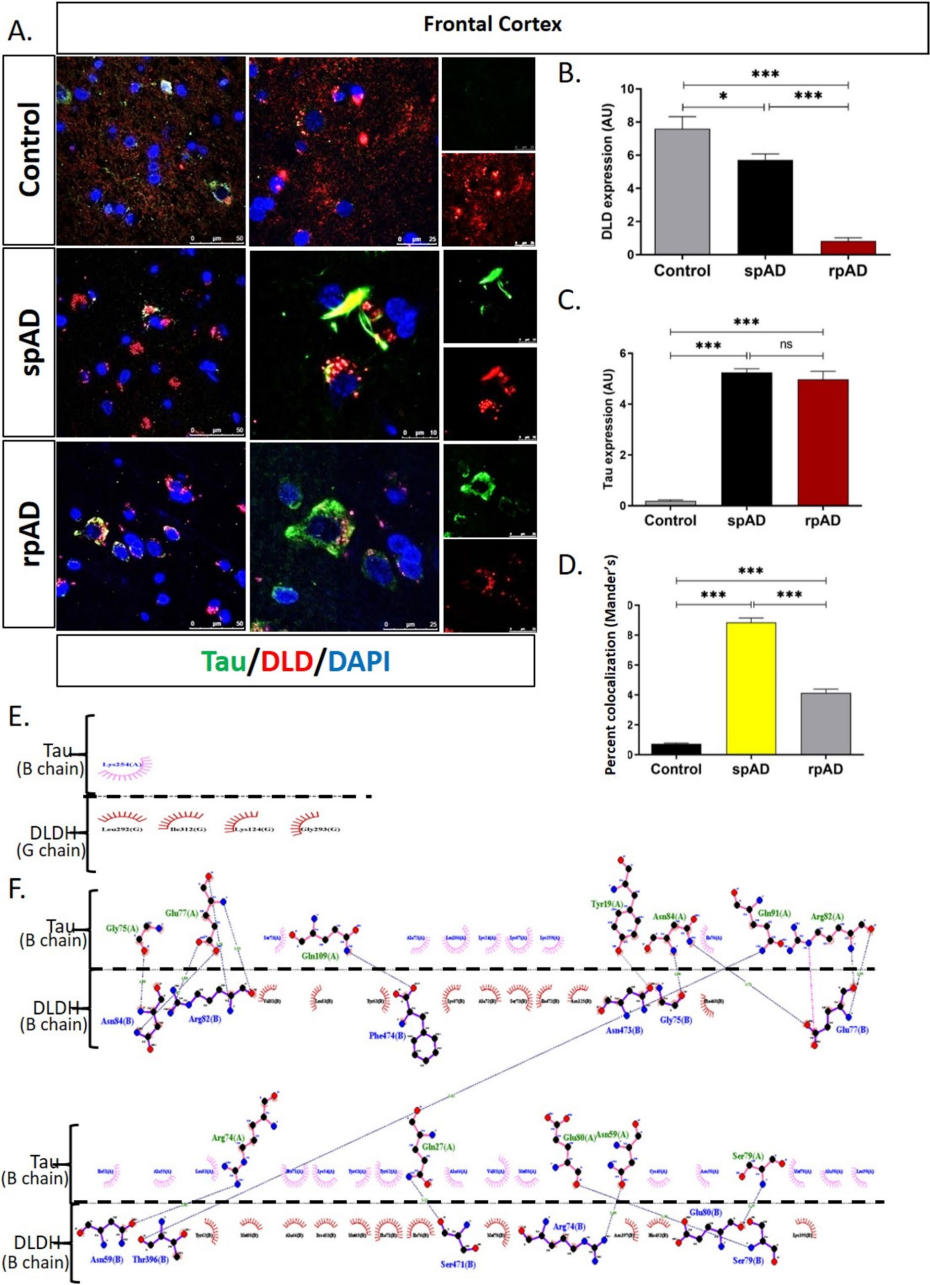
Colocalization of DLDH and Tau. Confocal immunofluorescence imaging (**A**) depicted the relative expression of DLDH (**B**) and Tau (**C**) in brain sections and a significantly higher colocalization between the two in spAD cases (**D**). Ligplot analysis revealed the residues involved in the interaction of Tau and DLD (**E**, **F**). The blue dotted line represents hydrogen bonding, and the red half-circles show DLDH hydrophobic residues. The pink half-circles show Tau hydrophobic residues. Data is presented as mean ± SD. “*” represents *p* < 0.05, “***” represents *p* < 0.001, ns = non-significant

**Fig. 5:**
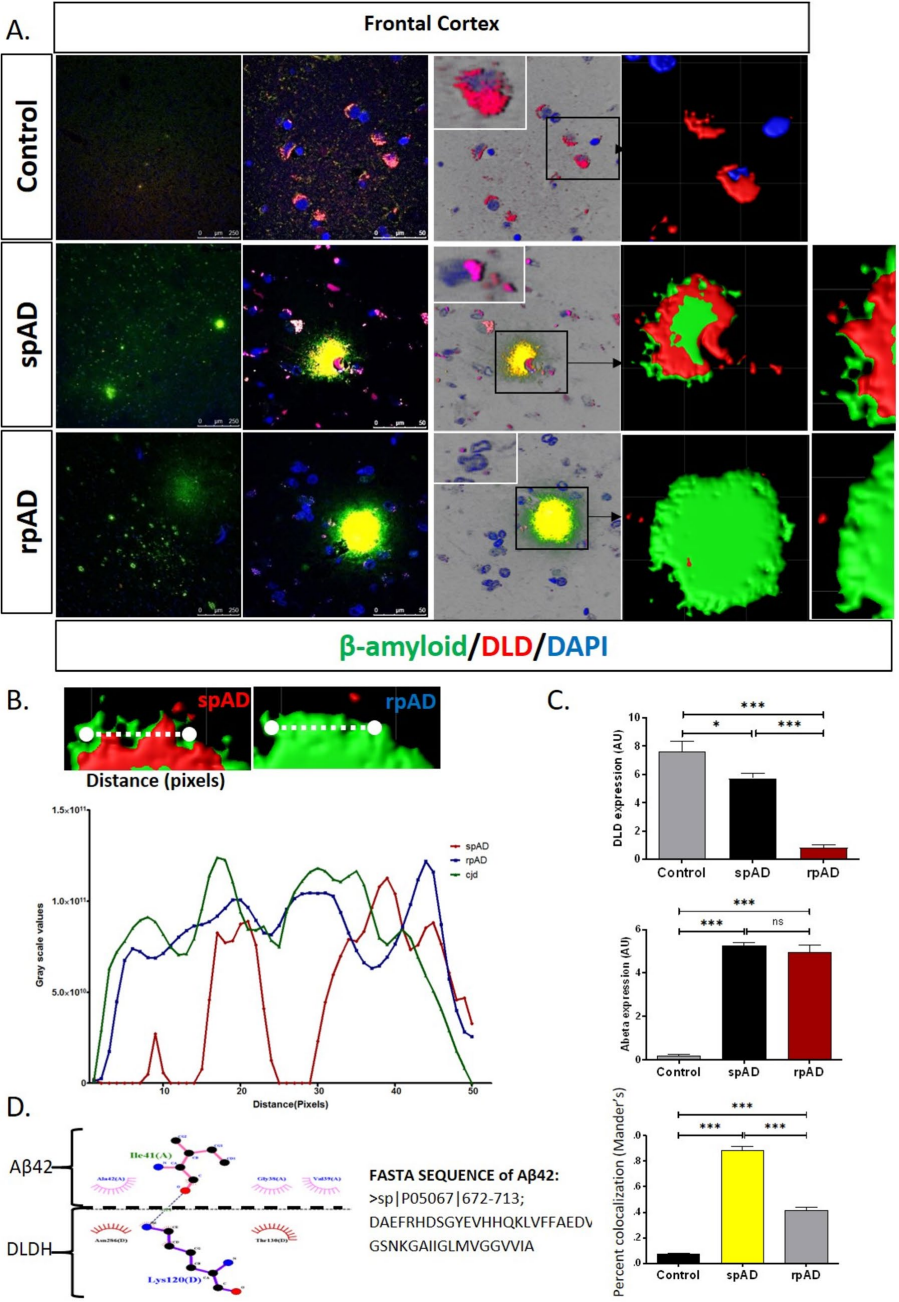
Colocalization of DLDH and Aβ. Confocal immunofluorescence imaging depicted the colocalization of DLDH and Aβ (**A**, **B**) in brain sections. A more extensive DLDH internalization in Aβ plaques was observed in rpAD in comparison to spAD brains. Ligplot analysis revealed the residues involved in the interaction of Tau and Aβ (**C**, **D**). The blue dotted line represents hydrogen bonding, and the red half-circles show DLDH hydrophobic residues. The pink half-circles show Tau hydrophobic residues

In situ evaluation of DLDH colocalization with the AD’s pathological proteins, i.e., Tau (Fig. 4) and Aβ (Fig. 5), was performed using confocal immunofluorescence imaging. This data also highlights a significant decrease in DLDH expression in rpAD brain tissues in comparison with spAD and controls. Additionally, total Tau expression significantly increased spAD and rpAD (Fig. 4C). Furthermore, the colocalization pattern of DLDH and Tau protein showed a significant increase in spAD in comparison with the rpAD frontal cortical region of the brain (Fig. 4D). Colocalization was significantly reduced in non-demented controls.

In silico analysis of Tau and DLDH complexes by Ligplot showed that the hydrophobic residues of DLDH (G chain) involved in its interaction are Leu292, Ile312, Lys124, and Gly293. The hydrophobic residue of Tau that is involved in interaction with DLDH (B chain) is Lys254 (Fig. 4E). Similarly, the hydrophobic residues of DLDH (B chain) that are involved in interaction with Tau are Val81, Leu83, Tyr63, Lys87, Ala72, Ser73, Ile472, Asn125, Phe468, Tyr62, Met88, Ala66, Pro453, Met65, Phe71, Ile76, Met78, Asn397, His452, and Lys395, and the hydrophobic residues of Tau that are involved in interaction with DLDH (B chain) are Ser73, Ala72, Leu106, Lys24, Lys87, Lys259, Ile76, Ile51, Ala55, Leu83, Phe71, Lys54, Tyr63, Tyr62, Ala66, Val81, Met88, Cys45, Asn58, Met78, Ala98, and Leu99. The functional groups involved in these interactions are presented in Supplementary Table 5.

Additionally, the colocalization pattern of DLDH on Aβ plaques in the cortex was also studied using confocal imaging (Fig. 5A). The high-resolution three-dimensional (3D) reconstruction revealed extensive DLDH internalization in Aβ plaques in rpAD compared with spAD frontal cortex brains (Fig. 5B). The hydrophobic residues of DLDH that are involved in interaction with Aβ42 are Asn286 and Thr130, and the hydrophobic residues of Aβ42 that are involved in interaction with DLDH are Ala42, Gly38, and Val39 (Fig. 5C, Supplementary Table 5).

### In Vitro Aggregation Assays

**Fig. 6:**
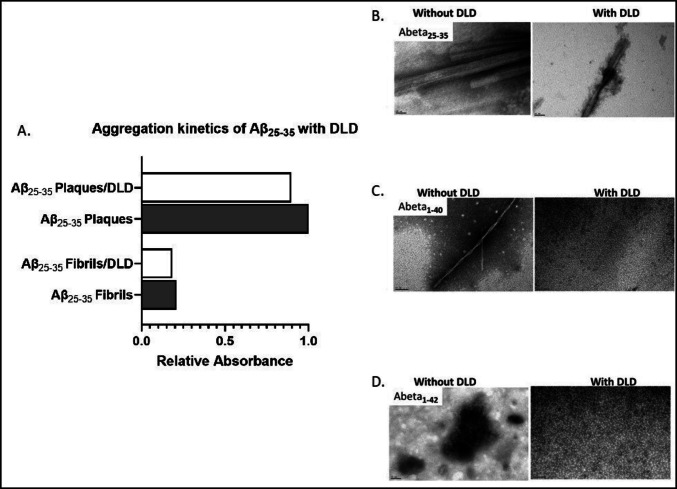
In vitro aggregation assays to identify the role of DLDH in Aβ aggregation. Addition of DLDH to the reaction mixture, optimized for the formation of Aβ fibrils, reduced the formation of fibrils as observed by ThT absorbance (**A**). Similar trends were observed for Aβ_25–35_ (**B**), Aβ_40_ (**C**), and Aβ_42_ (**D**) in negative staining electron microscopy. The plotted data represents two independent experiments

Owing to the differences in colocalization of DLDH and Aβ plaques, we studied the role of DLDH in the aggregation of Aβ (Fig. 6). The presence of DLDH in the reaction mixtures resulted in a reduced β-sheet formation within Aβ_25–35_ (selected due to its relevance to aggregation and neurotoxicity). Microscopic analysis further revealed that DLDH either prevented (Aβ_40_ and Aβ_42_) the formation of fibrils or reduced the rate of conversion of physiological Aβ into fibrils (Aβ_25–35_). A more extensive internalization of DLDH in Aβ plaques in rpAD may prevent it from inhibiting the conversion of physiological Aβ into amyloids, thereby leading to more neurotoxicity and a faster decline.

### Variations in the Levels of DLDH Throughout the Progression of AD

**Fig. 7:**
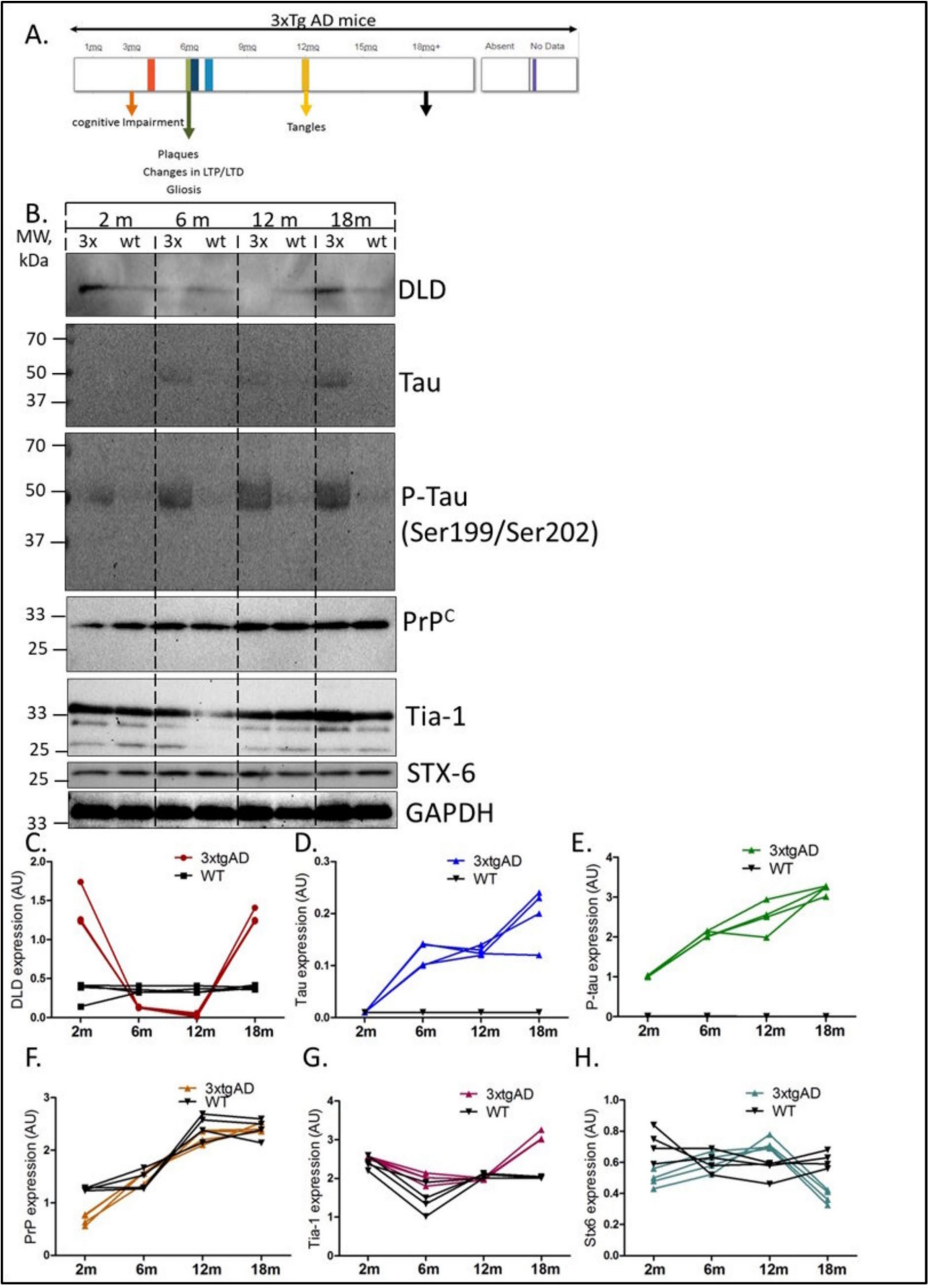
Expressional changes in DLDH and other relevant proteins during the progression of AD. 3xTg-AD mice models were developed, and proteins were extracted at 2, 6, 12, and 18 months for immunoblot analysis (**A**, **B**). The changes in relative expression of DLDH (**C**), Tau (**D**), P-Tau (**E**), PrP (**F**), Tia-1 (**G**), and syntaxin 6 (**H**) throughout the progression of the disease are depicted. “wt” stands for wild type

We monitored the expression of DLDH, Tau, P-Tau, PrP, Tia-1, and syntaxin 6 in 3xTg-AD mice at four different time points using Western blot analysis (Fig. 7) as an exploratory mechanistic evidence. These time points were selected according to the pathological changes in 3 × Tg-AD mice. DLDH expression was significantly higher than controls at a pre-symptomatic time point (2 months) and significantly downregulated at the age of the appearance of first behavioral abnormalities, Aβ plaques (6 months). Interestingly, the levels of DLDH significantly rise with hyperphosphorylated Tau at 12–18 months of the disease (Fig. 7B, C), and the expression of the Tau and P-Tau was significantly increased during the course of the disease from 2 to 18 months (Fig. 7A, B, D, E). However, PrP levels were significantly downregulated only at the age of first behavioral abnormalities at 6 months (Fig. 7B, F). The expression of Tia-1 (a marker associated with programmed cell death/apoptosis and that regulates alternative splicing of the gene encoding the Fas receptor, an apoptosis-promoting protein) showed a significant upregulation at 12–18 months of the disease (Fig. 7B, G). Furthermore, syntaxin 6, which is part of the family of target SNAREs and is involved in exporting and importing cell cargo through a process called cell trafficking, showed to be significantly upregulated at 12 months of hyperphosphorylated Tau expression of the disease and downregulated at 18 months of disease course (Fig. 7B, H).

### DLDH Interactors in rpAD and spAD

Identification of DLDH interacting partners was performed using co-immunoprecipitation. Upon the removal of common contaminants and the proteins that were found in binding controls, a total of 68 proteins were identified (Supplementary Table 6). Disease-specific analysis showed that 12 proteins (including clathrin coat assembly protein, alpha-crystallin, ubiquitin carboxyl-terminal hydrolase isozyme) were found in spAD cases only and were primarily involved in protein synthesis, transport, and folding. On the other hand, seven targets (including synaptogamin and syntaxin) were specific to rpAD and were primarily involved in neurotransmission and ion transport. The remaining proteins, common to multiple disease groups and controls, were involved in metabolism and synaptic transmission.

## Discusssion

Developing a comprehensive understanding of the pathological mechanisms involved in deriving various clinical variants of AD is crucial for developing diagnostic, prognostic, and therapeutic interventions. This information is especially lagging for the relatively newly discovered rpAD. Previous studies have reported that rpAD cases are not significantly different from spAD cases with respect to markers for inflammation and tissue damage or the distribution of plaques and tangles [36, 37]. However, APOE ε4 allelic frequency appears to be lower in rpAD cases in comparison to spAD [38, 39]. In the current dataset, it was also observed that the rpAD cases presented a significantly lower allelic frequency of ε4, while that of ε3 was significantly higher. Studies have identified ε4 as a potential risk factor for AD in contrast to the neutral and protective role of ε3 and ε2, respectively [40]. The findings highlight the subtle metabolic modifications underlying the rapid progression of AD.

In an attempt to comprehensively evaluate the proteomic changes underlying the two clinical variants of AD, we conducted a proteome-wide quantitative analysis. Previously, proteome evaluation of plaque-associated, RNA-binding, Ab-binding, and prion protein–binding fractions has been conducted in rpAD [5, 41–43]. However, none of these studies reported the changes in the whole proteome. We identified 79 common proteins in rpAD and spAD cases that showed significantly altered expression levels in comparison to controls. Moreover, 36 and 47 proteins showed specific regulatory levels in spAD and rpAD cases, respectively. A majority of differentially regulated proteins in rpAD were found to fall under the category of enzyme modulators, further highlighting the role of metabolic changes in rapid progression.

Among the differentially regulated proteins, the most profound change was evident in DLDH (13.4-fold change) in rpAD cases. DLDH is a key enzyme involved in the conversion of pyruvate to acetyl-CoA, which links glycolysis to the citric acid cycle and is vital for energy production from carbohydrates and fats [44, 45]. Additionally, DLDH can play both pro- and antioxidant roles, depending upon its conformation [14, 15]. Its key involvement in energy metabolism and oxidative stress modulation highlights its significance and potential pathological involvement in several disorders. Its aberrant expression has been previously reported in spAD, whereby its downregulation is associated with oxidative damage and pathogenesis [23, 24, 46]. The 13-fold reduction in its expression in rpAD, reported in the current study, highlights a significant disruption in energy metabolism and oxidative stress, both of which can have a drastic implication on the progression of AD. Our results were consistently similar across mass spectrometric and immunoblot evaluations.

Furthermore, evaluations on animal models of AD revealed that while most of the pathological proteins, i.e., Tau, phosphorylated-Tau, and prion, increased as the disease progressed, DLDH expressions were very high at the beginning of the disease and during the later stages. Its reduced expression, depicted in human rpAD patients, accompanied by a rapid cognitive decline, is hence a deviation from its normal expression in AD models. This deviation further highlights the significance of this pathway in rpAD. Prion, Tia-1, and syntaxin proteins were also evaluated in this experiment owing to their previously reported relation to rpAD [5, 43]. Evaluation of the DLDH interactome highlighted the involvement of proteins involved in transport and neurotransmission. Previous work has also suggested the involvement of these pathways in rpAD [41].

Owing to the gap in published literature, we were also interested in evaluating whether the expression differences were accompanied by variations in localization. We targeted two prominent pathological hallmarks of AD, i.e., Aβ and Tau. In rpAD, the colocalization pattern of DLDH and Tau protein showed a significant reduction in comparison to spAD, possibly due to its reduced expression in the former variant. The experiments also revealed an internalization of DLDH in the Aβ plaques, which may reduce the levels of DLDH available to perform physiological functions in the neuron. In silico evaluations reported an interaction between DLDH, Tau, and Aβ.

Other proteins that showed significant alteration in rpAD included deoxyuridine 5′-triphosphate nucleotidohydrolase, cytochrome c oxidase, dihydropteridine reductase, and haloacid dehalogenase–like hydrolase domain–containing protein 2; however, the difference, although significant, was not as profound as that observed for DLDH. Deoxyuridine 5′-triphosphate nucleotidohydrolase is involved in nucleotide metabolism and has previously been associated with neuroinflammation and AD [47]. The deficiency of mitochondrial metabolic protein, cytochrome c oxidase, has also been linked to AD [48]. Dihydropteridine reductase deficiency is associated with aberrations in the synthesis of neurotransmitters [49]. Aberrations in the expression of haloacid dehalogenase–like hydrolase domain–containing protein have been reported in hippocampal mitochondria extracted from animal models of AD [50].

Upon the evaluation of the expression, localization, interactions, and effects on aggregation, DLDH has emerged as a promising target underlying the pathogenesis of rpAD. Further studies on its diagnostic and therapeutic potential may provide useful outcomes for clinicians and AD patients.

## Conclusion

Our study provides comprehensive evidence that rpAD is characterized by distinct molecular and metabolic signatures compared to spAD, with pronounced alterations in mitochondrial function and energy metabolism. Quantitative proteomic profiling revealed a significant reduction in the expression of DLDH, a key mitochondrial enzyme, specifically in rpAD, which was further validated through biochemical and imaging analyses. The colocalization of DLDH with Aβ plaques and Tau aggregates, along with its impaired functional role in modulating Aβ aggregation, underscores its potential contribution to accelerated neurodegeneration in rpAD. Additionally, the altered interactome of DLDH in rpAD, enriched in neurotransmission and metabolic proteins, suggests a broader impact on neuronal homeostasis. Together, these findings identify DLDH as a central molecular discriminator of rpAD and provide a foundation for future diagnostic and therapeutic strategies targeting metabolic dysregulation in aggressive forms of AD.

## Supplementary Information

Below is the link to the electronic supplementary material.

Supplementary file 1 (PDF 285 KB)

Supplementary file 2 (PDF 355 KB)

## Data Availability

No datasets were generated or analysed during the current study.
