# Supplementary material for: Proteomic Profiling Reveals Mitochondrial Dysregulation in Rapidly Progressive Alzheimer’s: Role of DLDH in Amyloid Beta Aggregation
Source: Mol Neurobiol. 2025 Nov 19;63(1):73. doi: 10.1007/s12035-025-05327-0 (PMC12627125; doi:10.1007/s12035-025-05327-0)

Supplementary data 2DE. Comparative 2D-DIGE proteomic analysis of control and Alzheimer's disease samples. Representative two-dimensional gel electrophoresis (2-DE) maps of protein extracts from Ctrl (control), spAD (sporadic Alzheimer's disease), and rpAD (rapidly progressive Alzheimer's disease) are shown. Protein spots were separated according to their isoelectric point (pH 3–10, horizontal axis) and molecular weight (vertical axis, kDa). Differentially expressed protein spots are circled and numbered. The fused image (Ctrl + spAD + rpAD) highlights overlapping and distinct protein expression patterns, with color-coded spot variations indicating relative abundance differences between groups.

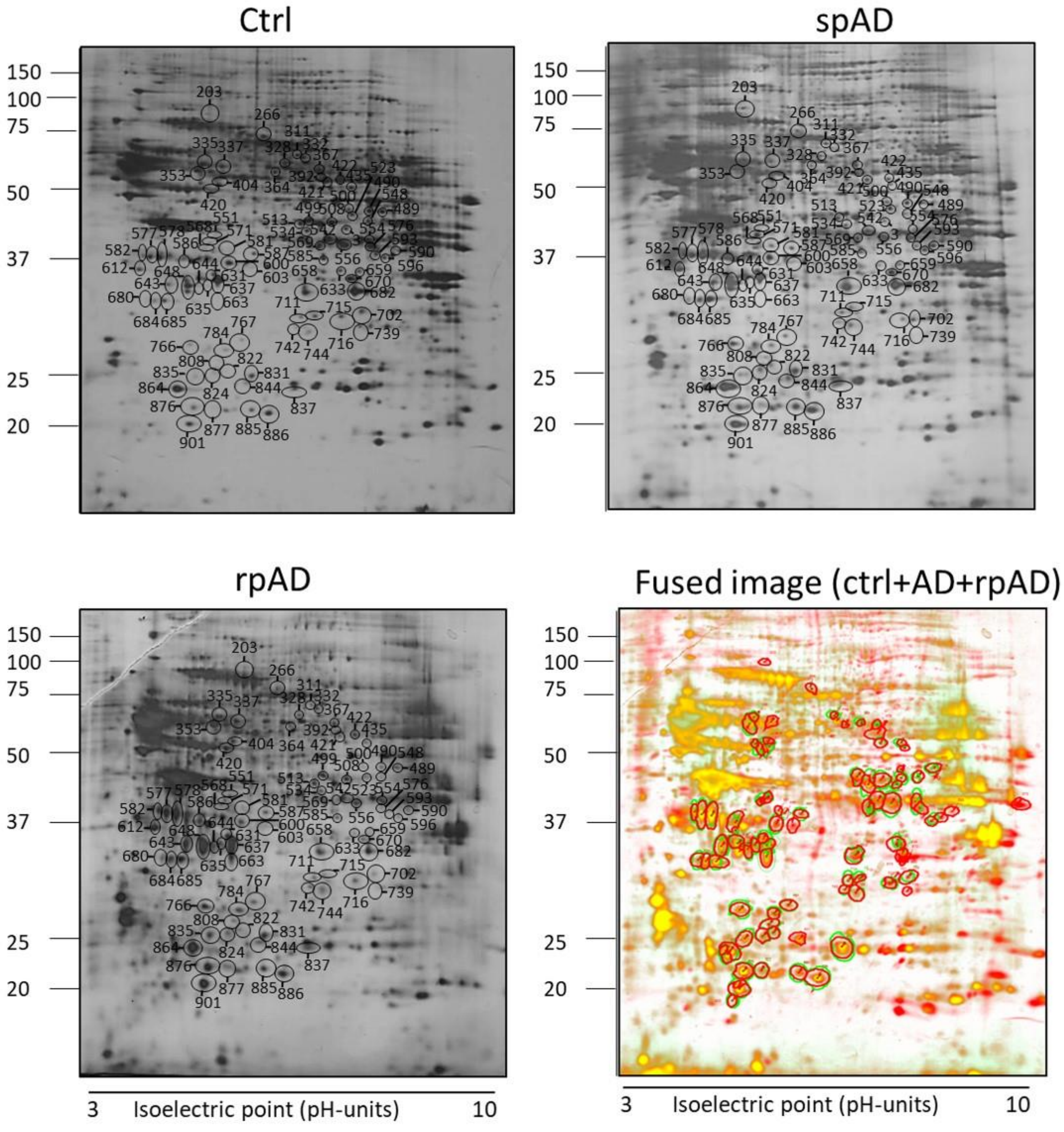

Supplement: Supplementary file 1 — (PDF 285 KB) [file 12035_2025_5327_MOESM1_ESM.pdf]
