# Supplementary material for: Proteomic Profiling Reveals Mitochondrial Dysregulation in Rapidly Progressive Alzheimer’s: Role of DLDH in Amyloid Beta Aggregation
Source: Mol Neurobiol. 2025 Nov 19;63(1):73. doi: 10.1007/s12035-025-05327-0 (PMC12627125; doi:10.1007/s12035-025-05327-0)
Supplement: Supplementary file 2 — (PDF 355 KB) [file 12035_2025_5327_MOESM2_ESM.pdf]

Supplementary Table 1: Allele and genotype frequency of ApoE among participants: Relative percentages of APOE alleles ε2, ε3 and ε4 in sAD, rpAD and ND control cases is depicted in this table.

**Genotype**	**spAD(n=14)**<br>**n (%)**	**rpAD (n=9)**<br>**n (%)**	**ND (n=10)**<br>**n (%)**
**Apo E Genotype**			
E2/2	1(7.1)	0(0)	2(20)
E2/3	1(7.1)	0(0)	3(30)
E3/2	0(0)	0(0)	0(0)
E3/3	3(21.4)	6(66.6)	4(40)
E3/4	9(64.2)	3(33.3)	1(10)
E2/4	0(0)	0(0)	0(0)
E4/4	0(0)	0(0)	0(0)
**Apo E Alleles**			
e2	3(11.5)	0	7(35)
e3	15(57.6)	15(83.3)	12(60)
e4	8(30.7)	3(16.6)	1(5)

Supplementary Table 2: List of differentially regulated proteins in both rpAD and spAD in comparison with control brain samples. Identifications were accepted, if established at a greater than 95.0% confidence while a minimum of two confident peptide identifications and a confidence threshold of 99.0% was required for protein identifications.

**Nr.**	**Protein name**	**Uniprot accession no.**	**Exclusive unique peptide count**	**Total spectrum count**	**Sequence coverage**	**Ctrl vs AD**	**Ctrl vs rpAD**
1	Actin-related protein 3	ARP3_HUMAN	10	37	25,80%	-2,6521443	2,700462
2	Fructose-bisphosphate aldolase C	ALDOC_HUMAN	8	11	19,00%	-3,8208891	-3,0194407
3	LanC-like protein 1	LANC1_HUMAN	9	14	21,80%	-3,8208891	-3,0194407
4	Glutamine synthetase	GLNA_HUMAN	7	43	16,90%	-5,3385471	-2,5565804
5	Fructose-bisphosphate aldolase C	ALDOC_HUMAN	12	36	30,50%	-8,0135977	-2,5565804
6	Mitogen-activated protein kinase 1	MK01_HUMAN	4	17	20,60%	-2,0630865	-11,257188
7	Fructose-bisphosphate aldolase C	ALDOC_HUMAN	8	17	29,70%	-2,0630865	-11,257188
8	Aspartate aminotransferase, cytoplasmic	AATC_HUMAN	8	20	18,60%	-2,0630865	-11,257188
9	10 kDa heat shock protein, mitochondrial	CH10_HUMAN	3	4	29,40%	2,0034986	2,3316443
10	40S ribosomal protein S11	RS11_HUMAN	3	3	16,50%	2,0034986	2,3316443
11	40S ribosomal protein S19	RS19_HUMAN	3	3	19,30%	2,0034986	2,3316443
12	40S ribosomal protein S3	RS3_HUMAN	5	5	23,00%	2,0034986	2,3316443
13	40S ribosomal protein S8	RS8_HUMAN	3	6	18,80%	2,0034986	2,3316443
14	40S ribosomal protein S9	RS9_HUMAN	4	5	20,10%	2,0034986	2,3316443
15	40S ribosomal protein SA	RSSA_HUMAN	5	7	23,10%	2,0034986	2,3316443
16	60S ribosomal protein L17	RL17_HUMAN	4	5	26,60%	2,0034986	2,3316443
17	78 kDa glucose-regulated protein	GRP78_HUMAN	11	22	26,00%	2,0034986	2,3316443

18	Actin, cytoplasmic 1	ACTB_HUMAN	3	28	28,00%	2,0034986	2,3316443
19	Alpha-enolase	ENOA_HUMAN	6	16	22,80%	2,0034986	2,3316443
20	Alpha-soluble NSF attachment protein 3	SNAA_HUMAN	5	8	18,60%	2,0034986	2,3316443
21	ATP synthase subunit alpha, mitochondrial	ATPA_HUMAN	7	9	15,90%	2,0034986	2,3316443
22	ATP synthase subunit beta, mitochondrial	ATPB_HUMAN	8	12	19,10%	2,0034986	2,3316443
23	Elongation factor 1-alpha 1	EF1A1_HUMAN	6	13	16,50%	2,0034986	2,3316443
24	Endoplasmin	ENPL_HUMAN	13	19	18,10%	2,0034986	2,3316443
25	Eukaryotic translation initiation factor 5A-1	IF5A1_HUMAN	3	5	18,20%	2,0034986	2,3316443
26	Heat shock cognate 71 kDa protein	HSP7C_HUMAN	8	24	26,50%	2,0034986	2,3316443
27	Heat shock protein HSP 90-alpha	HS90A_HUMAN	6	29	17,10%	2,0034986	2,3316443
28	Heat shock protein HSP 90-beta	HS90B_HUMAN	7	29	20,00%	2,0034986	2,3316443
29	Heterogeneous nuclear ribonucleoprotein A3	ROA3_HUMAN	4	4	15,10%	2,0034986	2,3316443
30	Heterogeneous nuclear ribonucleoprotein K	HNRPK_HUMAN	7	8	19,70%	2,0034986	2,3316443
31	Heterogeneous nuclear ribonucleoproteins A2/B1	ROA2_HUMAN	6	7	21,00%	2,0034986	2,3316443
32	Malate dehydrogenase, mitochondrial	MDHM_HUMAN	5	5	18,60%	2,0034986	2,3316443
33	Nucleophosmin	NPM_HUMAN	5	9	24,10%	2,0034986	2,3316443
34	Nucleoside diphosphate kinase A	NDKA_HUMAN	3	5	22,40%	2,0034986	2,3316443
35	Phosphoglycerate kinase 1	PGK1_HUMAN	5	5	16,30%	2,0034986	2,3316443
36	Protein SET	SET_HUMAN	5	8	22,80%	2,0034986	2,3316443
37	Ras-related protein Rab-1A	RAB1A_HUMAN	3	4	21,00%	2,0034986	2,3316443

38	Tubulin alpha-1A chain	TBA1A_HUMAN	3	33	33,90%	2,0034986	2,3316443
39	Tubulin beta chain	TBB5_HUMAN	3	27	33,10%	2,0034986	2,3316443
40	Ubiquitin-60S ribosomal protein L40	RL40_HUMAN	4	11	33,60%	2,0034986	2,3316443
41	Voltage-dependent anion-selective channel protein 1	VDAC1_HUMAN	4	5	18,40%	2,0034986	2,3316443
42	Hemoglobin subunit beta	HBB_HUMAN	4	7	34,70%	-2,5439395	-2,9541206
43	Ig gamma-1 chain C region	IGHG1_HUMAN	3	15	25,20%	-2,5439395	-2,9541206
44	Isocitrate dehydrogenase [NAD] subunit alpha, mitochondrial	IDH3A_HUMAN	14	46	28,10%	-2,5439395	-2,9541206
45	NAD-dependent protein deacetylase sirtuin-2	SIR2_HUMAN	6	18	17,20%	-2,5439395	-2,9541206
46	Serum albumin	ALBU_HUMAN	22	63	33,00%	-2,5439395	-2,9541206
47	Transaldolase	TALDO_HUMAN	7	15	21,40%	-2,5439395	-2,9541206
48	Ubiquitin-60S ribosomal protein L40	RL40_HUMAN	3	6	29,70%	-2,9372767	-10,469919
49	Cystatin-A	CYTA_HUMAN	4	4	54,10%	-2,9372767	-10,469919
50	Nitrilase homolog 1	NIT1_HUMAN	6	8	20,80%	-2,9372767	-10,469919
51	Voltage-dependent anion-selective channel protein 2	VDAC2_HUMAN	7	10	24,80%	-2,9372767	-10,469919
52	Malate dehydrogenase, cytoplasmic	MDHC_HUMAN	11	28	32,30%	-5,2000939	-3,3492664
53	Glycerol-3-phosphate dehydrogenase 1-like protein	GPD1L_HUMAN	10	23	31,60%	-3,197459	-15,460751
54	Phytanoyl-CoA hydroxylase-interacting protein	PHYIP_HUMAN	7	22	26,70%	-4,5981781	-5,6248452
55	Histone H4	H4_HUMAN	3	4	27,20%	3,2655572	5,4273999
56	Prohibitin	PHB_HUMAN	15	81	52,90%	3,2655572	5,4273999
57	40S ribosomal protein S10	RS10_HUMAN	4	5	20,00%	2,267513	3,0779869
58	60S ribosomal protein L17	RL17_HUMAN	3	3	17,90%	2,267513	3,0779869

59	60S ribosomal protein L18	RL18_HUMAN	4	6	25,00%	2,267513	3,0779869
60	60S ribosomal protein L23a	RL23A_HUMAN	3	4	16,00%	2,267513	3,0779869
61	60S ribosomal protein L24	RL24_HUMAN	3	5	18,50%	2,267513	3,0779869
62	60S ribosomal protein L26	RL26_HUMAN	3	3	17,20%	2,267513	3,0779869
63	ATP synthase subunit O, mitochondrial	ATPO_HUMAN	4	5	22,50%	2,267513	3,0779869
64	Cofilin-1	COF1_HUMAN	5	12	43,40%	2,267513	3,0779869
65	Enoyl-CoA hydratase, mitochondrial	ECHM_HUMAN	10	28	36,90%	2,267513	3,0779869
66	Heat shock protein beta-1	HSPB1_HUMAN	9	17	42,00%	2,267513	3,0779869
67	Membrane-associated progesterone receptor component 1	PGRC1_HUMAN	3	3	21,50%	2,267513	3,0779869
68	Peptidyl-prolyl cis-trans isomerase B	PPIB_HUMAN	7	13	33,80%	2,267513	3,0779869
69	Peroxiredoxin-1	PRDX1_HUMAN	7	25	46,20%	2,267513	3,0779869
70	Peroxiredoxin-2	PRDX2_HUMAN	6	20	32,80%	2,267513	3,0779869
71	Peroxiredoxin-6	PRDX6_HUMAN	6	7	22,30%	2,267513	3,0779869
72	Phosphatidylethanolamine-binding protein 1	PEBP1_HUMAN	3	9	22,50%	2,267513	3,0779869
73	Proteasome subunit beta type-5	PSB5_HUMAN	4	5	18,60%	2,267513	3,0779869
74	Ras-related protein Rab-1A	RAB1A_HUMAN	4	6	26,80%	2,267513	3,0779869
75	Ras-related protein Rab-2A	RAB2A_HUMAN	3	5	17,90%	2,267513	3,0779869
76	Ras-related protein Rab-7a	RAB7A_HUMAN	4	8	23,20%	2,267513	3,0779869
77	Redox-regulatory protein FAM213A	F213A_HUMAN	4	8	21,00%	2,267513	3,0779869
78	Transgelin-3	TAGL3_HUMAN	4	6	22,60%	2,267513	3,0779869
79	Ubiquitin-conjugating enzyme E2 K	UBE2K_HUMAN	3	3	16,50%	2,267513	3,0779869

Supplementary Table 3: List of differentially regulated proteins in spAD in comparison with control and rpAD brain samples. Identifications were accepted, if established at a greater than 95.0% confidence while a minimum of two confident peptide identifications and a confidence threshold of 99.0% was required for protein identifications.

Nr.	Protein name	Uniprot Accession no.	Exclusive unique peptide count	Total spectrum count	Sequence coverage	Fold change
1	14-3-3 protein sigma	1433S_HUMAN	3	6	15,70%	-6,61578401
2	60 kDa heat shock protein, mitochondrial	CH60_HUMAN	18	44	36,60%	-5,02630195
3	Alcohol dehydrogenase class-3	ADHX_HUMAN	10	33	20,10%	-3,97036422
4	Alpha-internexin	AINX_HUMAN	13	28	29,30%	-5,38677951
5	Bifunctional purine biosynthesis protein PURH	PUR9_HUMAN	11	14	26,20%	-5,02630195
6	C-1-tetrahydrofolate synthase, cytoplasmic	C1TC_HUMAN	18	24	23,00%	-2,88796012
7	Cornifin-B	SPR1B_HUMAN	1	6	52,80%	-2,0864397
8	D-3-phosphoglycerate dehydrogenase	SERA_HUMAN	8	14	15,90%	-2,06170599
9	Diphosphoinositol polyphosphate phosphohydrolase 1	NUDT3_HUMAN	3	4	19,20%	-2,0864397
10	E3 ubiquitin-protein ligase CHIP	CHIP_HUMAN	8	12	24,10%	-2,32974539
11	Gamma-glutamylcyclotransferase	GGCT_HUMAN	4	5	23,90%	-2,0864397
12	Gamma-soluble NSF attachment protein	SNAG_HUMAN	5	7	15,40%	-5,02630195
13	Glucosamine-6-phosphate isomerase 1	GNPI1_HUMAN	9	35	34,90%	-3,77917248
14	Glutathione S-transferase P	GSTP1_HUMAN	3	7	17,10%	2,77045455

15	Glyceraldehyde-3-phosphate dehydrogenase	G3P_HUMAN	4	6	16,10%	-2,0864397
16	GMP reductase 2	GMPR2_HUMAN	5	17	17,50%	-3,97036422
17	Heat shock-related 70 kDa protein 2	HSP72_HUMAN	3	17	16,10%	-2,32974539
18	Histone H2B type 1-C/E/F/G/I	H2B1C_HUMAN	0	5	34,90%	-6,61578401
19	Histone H2B type 3-B	H2B3B_HUMAN	0	4	27,80%	-6,61578401
20	Junction plakoglobin	PLAK_HUMAN	15	31	22,40%	-6,61578401
21	Keratinocyte proline-rich protein1	KPRP_HUMAN	9	21	20,40%	-2,0864397
22	Microtubule-associated protein RP/EB family member 2	MARE2_HUMAN	4	10	15,00%	-5,02630195
23	Myelin proteolipid protein	MYPR_HUMAN	4	4	15,50%	2,77045455
24	Myristoylated alanine-rich C-kinase substrate	MARCS_HUMAN	5	5	28,30%	-5,02630195
25	Neurofilament medium polypeptide	NFM_HUMAN	19	68	19,00%	-5,38677951
26	Poly [ADP-ribose] polymerase 1	PARP1_HUMAN	14	21	16,90%	-2,88796012
27	Protein DJ-1 OS=Homo sapiens	PARK7_HUMAN	7	21	32,80%	2,77045455
28	Protein S100-A7 OS=Homo sapiens	S10A7_HUMAN	3	3	35,60%	-2,0864397
29	Protein S100-A8 OS=Homo sapiens	S10A8_HUMAN	2	6	23,70%	-2,0864397
30	Pyridoxal kinase OS=Homo sapiens	PDXK_HUMAN	6	12	23,40%	-6,61578401

31	Pyruvate kinase PKM OS=Homo sapiens	KPYM_HUMAN	15	30	33,00%	-5,02630195
32	T-complex protein 1 subunit alpha	TCPA_HUMAN	8	14	18,30%	-5,02630195
33	T-complex protein 1 subunit beta	TCPB_HUMAN	9	10	20,00%	-2,06170599
34	T-complex protein 1 subunit beta	TCPB_HUMAN	9	10	20,00%	-2,06170599
35	Ubiquitin-like modifier-activating enzyme 1	UBA1_HUMAN	19	36	21,70%	-2,88796012
36	V-type proton ATPase subunit B, brain isoform	VATB2_HUMAN	9	108	20,00%	-5,38677951

Supplementary Table 4: List of differentially regulated proteins in rpAD in comparison with control and spAD brain samples. Identifications were accepted, if established at a greater than 95.0% confidence while a minimum of two confident peptide identifications and a confidence threshold of 99.0% was required for protein identifications.

**Nr.**	**Protein name**	**Uniprot accession no.**	**Exclusive unique peptide count**	**Total spectrum count**	**Sequence coverage**	**Fold change**
1	40S ribosomal protein S28	RS28_HUMAN	2	6	30,40%	2,7191263
2	40S ribosomal protein S30	RS30_HUMAN	2	4	18,60%	2,7191263
3	60S ribosomal protein L35a	RL35A_HUMAN	2	4	19,10%	2,7191263
4	60S ribosomal protein L38	RL38_HUMAN	4	4	48,60%	2,7191263
5	6-phosphogluconolactonase	6PGL_HUMAN	5	29	24,40%	5,47707559

6	Acylphosphatase-1	ACYP1_HUMAN	2	3	23,20%	2,7191263
7	Adaptin ear-binding coat-associated protein 1	NECP1_HUMAN	4	7	17,80%	2,2820296
8	Antileukoproteinase	SLPI_HUMAN	3	4	15,90%	-4,58501441
9	Apolipoprotein E	APOE_HUMAN	5	6	18,60%	2,44590965
10	ATP synthase subunit beta, mitochondrial	ATPB_HUMAN	8	12	19,10%	2,33164435
11	ATP synthase subunit g, mitochondrial	ATP5L_HUMAN	2	3	23,30%	2,7191263
12	ATPase inhibitor, mitochondrial	ATIF1_HUMAN	2	4	15,10%	2,7191263
13	Beta-soluble NSF attachment protein	SNAB_HUMAN	13	81	53,40%	2,44590965
14	BolA-like protein 2	BOLA2_HUMAN	2	7	29,10%	2,7191263
15	F-actin-capping protein subunit alpha-2	CAZA2_HUMAN	3	4	14%	1,679584261
16	Cellular retinoic acid-binding protein 1	RABP1_HUMAN	7	20	41,60%	2,7191263

17	Cellular retinoic acid-binding protein 2	RABP2_HUMAN	3	12	23,20%	2,7191263
18	Charged multivesicular body protein 2a	CHM2A_HUMAN	5	7	19,80%	2,7191263
19	Cytochrome c	CYC_HUMAN	4	8	32,40%	2,7191263
20	Cytochrome c oxidase subunit 5B, mitochondrial	COX5B_HUMAN	3	3	24,80%	2,7191263
21	D-dopachrome decarboxylase-like protein	DDTL_HUMAN	2	5	15,70%	2,7191263
22	Deoxyuridine 5'-triphosphate nucleotidohydrolase, mitochondrial	DUT_HUMAN	4	5	19,40%	3,07798685
23	Dihydrolipoyl dehydrogenase, mitochondrial	DLDH_HUMAN	10	16	19,30%	-13,4101077
24	Dihydropteridine reductase	DHPR_HUMAN	6	20	37,70%	2,27970231
25	Dynein light chain roadblock-type 1	DLRB1_HUMAN	1	3	29,20%	2,7191263
26	Haloacid dehalogenase-like hydrolase domain-containing protein 2	HDHD2_HUMAN	4	11	16,60%	2,7191263

27	Haptoglobin	HPT_HUMAN	5	6	17,70%	2,2820296
28	Heterogeneous nuclear ribonucleoproteins C1/C2	HNRPC_HUMAN	5	6	18,60%	3,4196847
29	Hypoxanthine-guanine phosphoribosyltransferase	HPRT_HUMAN	9	41	45,00%	2,27970231
30	Ig alpha-2 chain C region	IGHA2_HUMAN	1	7	19,40%	-4,58501441
31	Myotrophin	MTPN_HUMAN	2	6	22,00%	2,7191263
32	Neutrophil defensin 1	DEF1_HUMAN	3	3	20,20%	-4,58501441
33	Prefoldin subunit 6	PFD6_HUMAN	2	3	15,50%	2,7191263
34	Profilin-1	PROF1_HUMAN	5	7	48,60%	2,7191263
35	Profilin-2	PROF2_HUMAN	3	8	27,90%	2,7191263
36	Programmed cell death protein 5	PDCD5_HUMAN	2	4	19,20%	2,7191263
37	Protein disulfide-isomerase A3	PDIA3_HUMAN	15	34	32,50%	5,08157303
38	Prothymosin alpha	PTMA_HUMAN	5	13	23,40%	2,7191263
39	Putative peptidyl-tRNA hydrolase PTRHD1	PTRD1_HUMAN	2	3	15,00%	2,7191263

40	Pyruvate dehydrogenase E1 component subunit beta, mitochondrial	ODPB_HUMAN	5	5	17,00%	3,4019836
41	S-formylglutathione hydrolase	ESTD_HUMAN	8	29	34,80%	-2,16658517
42	SH3 domain-binding glutamic acid-rich-like protein 3	SH3L3_HUMAN	4	5	39,80%	2,7191263
43	Spermidine synthase	SPEE_HUMAN	6	14	25,20%	2,44590965
44	Thioredoxin	THIO_HUMAN	2	3	17,10%	2,7191263
45	Transcription elongation factor B polypeptide 1	ELOC_HUMAN	4	10	45,50%	2,7191263
46	Triosephosphate isomerase	TPIS_HUMAN	9	13	41,30%	2,27970231
47	Tubulin-specific chaperone A	TBCA_HUMAN	5	7	36,10%	2,7191263
48	U6 snRNA-associated Sm-like protein LSm2	LSM2_HUMAN	2	3	31,60%	2,7191263

Supplementary Table 5: Atoms and functional groups involved in DLD interactions. The amino acid residues of DLDH, involved in the interactions with Tau and Aβ, and their respective groups have been enlisted. The distance between interacting partners is presented in Angstrom units.

**DLDH residues**	**Atoms involved**	**Aβ42 residues**	**Atoms involved**	**Distance (Å)**
Lys 120 (D)	NZ	Ile 41	O	3.21
**DLDH residues**	**Atoms involved**	**Tau residues**	**Atoms involved**	**Distance (Å)**
Asn 84 (B)	N	Gly 75	O	2.80
Asn 84 (B)	ND2	Glu 77	OE1	2.67
Arg 82 (B)	NH1	Glu 77	OE2	2.81
Arg 82 (B)	CA	Glu 77	O	2.76
Arg 82 (B)	O	Glu 77	N	2.95
Phe 474 (B)	OXT	Gln 109	NE2	3.11
Asn 473 (B)	OD1	Tyr19	OH	2.90
Gly 75 (B)	O	Asn 84	N	2.86
Glu 77 (B)	OE2	Asn 84	ND2	2.75
Glu 77 (B)	O	Arg 82	N	2.81
Glu 77 (B)	N	Arg 82	O	2.99
Asn 59 (B)	OD1	Arg 74	NH1	3.03
Thr 396 (B)	O	Gln 91	NE2	3.13
Ser 471 (B)	O	Gln 27	NE2	3.32
Arg 74 (B)	NH1	Asn 59	OD1	3.09
Glu 80 (B)	O	Ser 79	N	3.29
Ser 79 (B)	N	Glu 80	O	3.26

Supplementary Table 6: List of DLDH Interactors in spAD, rpAD, sCJD and controls. eptides were identified from 3 pooled biological replicates per experimental group and identifications were accepted, if established at a greater than 95.0% confidence while a minimum of two confident peptide identifications and a confidence threshold of 99.0% was required for protein identifications. The localization and functional category of identified DLDH interactors was annotated using UniProtKB database.

**Nr.**	**Identified Proteins**	**Accession Number**	**Specificity**	**Subcellular Localization**	**Functional pathways**
1	Cluster of Serine/threonine-protein phosphatase 2A 65 kDa regulatory subunit A alpha isoform	2AAA_HUMAN	AD	Cytoplasm	Chromosome partition
2	Aspartate aminotransferase, mitochondrial	AATM_HUMAN	AD, CJD	Mitochondria and cell membrane	Lipid Transport
3	Acyl-coenzyme A thioesterase 13	ACO13_HUMAN	CJD	Nucleus, Cytosol, Mitochondria	Acyl-CoA hydrolase
4	Aconitate hydratase, mitochondrial	ACON_HUMAN	CJD	Mitochondria	Tricarboxylic acid cycle
5	Activator of 90 kDa heat shock protein ATPase homolog 1	AHSA1_HUMAN	C, AD	Cytosol	Stress response
6	Clathrin coat assembly protein AP181	AP180_HUMAN	AD	Cell membrane	Protein transport, Transport
7	AP-2 complex subunit mu	AP2M1_HUMAN	CJD	Cell membrane	Protein transport, Transport
8	Arginase-1	ARGI1_HUMAN	AD, rpAD	Cytoplasm	Adaptive Immunity and Urea cycle
9	Sodium/potassium-transporting ATPase subunit beta-1	AT1B1_HUMAN	C,AD	Membrane	Ion transport
10	Plasma membrane calcium-transporting ATPase 2	AT2B2_HUMAN	rpAD	Cell membrane	Ion transport
11	ATP synthase subunit O, mitochondrial	ATPO_HUMAN	CJD	Mitochondria	Ion transport
12	Bleomycin hydrolase	BLMH_HUMAN	C, rpAD	Cytoplasm	Proteolysis
13	BPI fold-containing family A member 1	BPIA1_HUMAN	C,AD,rpAD	Secreted	Immunity, Innate immunity
14	Cullin-associated NEDD8-dissociated protein 1	CAND1_HUMAN	AD	Nucleus and cytoplasm	Differentiation

15	Carbonyl reductase [NADPH] 1	CBR1_HUMAN	C	Cytoplasm	Oxidoreductase
16	Cytosolic non-specific dipeptidase	CNDP2_HUMAN	C	Cytoplasm	Peptidase activity
17	Cornulin	CRNN_HUMAN	C	Cytoplasm	Cell proliferation
18	Alpha-crystallin B chain	CRYAB_HUMAN	AD	Nucleus	Chaperone
19	Ketimine reductase mu-crystallin	CRYM_HUMAN	C,AD,rpAD	Cytoplasm	Oxidoreductase
20	Versican core protein	CSPG2_HUMAN	C, AD	Extracellular matrix	Adhesion, CNS development
21	Cysteine and glycine-rich protein 1	CSRP1_HUMAN	CJD	Nucleus	Platelet aggregation
22	Dihydropteridine reductase	DHPR_HUMAN	C, AD	Mitochondria and cytosol	Amino acid metabolism
23	Dihydropyrimidinase-related protein 3	DPYL3_HUMAN	C, AD	Cytoplasm	Neuronal growth
24	Desmocollin-1	DSC1_HUMAN	C,AD,rpAD	Cell membrane	Cell adhesion
25	Trifunctional enzyme subunit beta	ECHB_HUMAN	CJD	Endoplamic Reticulum	Fatty acid beta-oxidation
26	Extracellular matrix protein 2	ECM1_HUMAN	rpAD	Secreted	Mineral balance, Osteogenesis
27	F-box only protein 51	FBX50_HUMAN	rpAD	Cytoplasm	Cell proliferation
28	Alpha-2-HS-glycoprotein	FETUA_HUMAN	rpAD	Cytoplasm	Mineral balance
29	Glutathione reductase	GSHR_HUMAN	CJD	Mitochondria	Cell redox homeostasis
30	Hemoglobin subunit alpha	HBA_HUMAN	CJD	Cytosol	Oxygen transport
31	Isocitrate dehydrogenase subunit alpha	IDH3A_HUMAN	C, AD	Mitochondria	Tricarboxylic acid cycle

32	Lysosome-associated membrane glycoprotein 2	LAMP1_HUMAN	AD	Lysosome	Neutrophil degranulation
33	Protein LEG1 homolog	LEG1H_HUMAN	C	Secreted	Development
34	Loricrin	LORI_HUMAN	rpAD	Cytoplasm	Keratinization
35	Myelin-associated glycoprotein	MAG_HUMAN	AD	Cell membrane	Cell adhesion
36	Microtubule-associated protein 1A	MAP1A_HUMAN	AD, CJD	Cytoskeleton	Cytoskeleton organization
37	Microtubule-associated protein 1B	MAP1B_HUMAN	AD, CJD	Cytoskeleton	Axon extension
38	Myelin basic protein	MBP_HUMAN	C, CJD	Nucleus	Synaptic Transmission
39	Methylmalonate-semialdehyde dehydrogenase	MMSA_HUMAN	CJD	Mitochondria	Pyrimidine metabolism
40	Myelin P2 protein	MYP2_HUMAN	CJD	Cytoplasm	Lipid Transport
41	Dihydrolipoyllysine-residue acetyltransferase component of pyruvate dehydrogenase complex, mitochondrial	ODP2_HUMAN	C, AD	Mitochondria	Glucose metabolism
42	Pyruvate dehydrogenase E1 component subunit alpha, somatic form, mitochondrial	ODPB_HUMAN	C,AD,rpAD	Mitochondria	Carbohydrate metabolism
43	Protein kinase C and casein kinase substrate in neurons protein 1	PACN1_HUMAN	AD	Cytoplasm and cell membrane	Endocytosis
44	Serum paraoxonase/arylesterase 1	PON1_HUMAN	C,AD,rpAD	Extracellular matrix	Hydrolysis
45	Profilin-2	PROF2_HUMAN	C, AD, rpAD	Cytoskeleton	Protein stablization
46	Cluster of Ras-related protein Rap-2a	RAP2A_HUMAN	C, rpAD	Endosome	Protein localization
47	2-iminobutanoate/2-iminopropanoate deaminase	RIDA_HUMAN	CJD	Nucleus and Mitochondria	Deaminase
48	60S ribosomal protein L12	RL12_HUMAN	C	Cytosol, Nucleus	Translation

49	60S ribosomal protein L18	RL18_HUMAN	AD	Cytosol, Nucleus	Translation
50	60 kDa SS-A/Ro ribonucleoprotein	RO60_HUMAN	C,AD,rpAD	Cytoplasm	Transcription and Signalling
51	40S ribosomal protein S19	RS19_HUMAN	CJD	Nucleus	Translation, rRNA processing
52	40S ribosomal protein S20	RS20_HUMAN	CJD	Cytoplasm	Translation
53	Secernin-1	SCRN1_HUMAN	AD	Cytoplasm	Exocytosis
54	Single-stranded DNA-binding protein, mitochondrial	SSBP_HUMAN	AD	Mitochondria	DNA replication
55	Syntaxin-1B	STX1B_HUMAN	rpAD	Cytoskeleton and nucleus	Neurotransmission
56	Synaptic vesicle glycoprotein 2A	SV2A_HUMAN	C,AD,rpAD	Synapse	Neurotransmission
57	Synapsin-1	SYN1_HUMAN	AD, CJD	Synapse	Neurotransmission
58	Synapsin-2	SYN2_HUMAN	C,AD,rpAD	Synapse	Synaptic transmission
59	Synaptotagmin-1	SYT1_HUMAN	rpAD	Synaptic vesicle membrane and cytoplasm	Synaptic transmission and brain development
60	Microtubule-associated protein tau	TAU_HUMAN	C, AD, CJD	Cytosol	Caspase-mediated cleavage of cytoskeletal proteins
61	Transcobalamin-1	TCO1_HUMAN	CJD	Secreted	Ion transport
62	Tenascin-R	TENR_HUMAN	Con	Extracellular matrix	Cell adhesion
63	Protein-glutamine gamma-glutamyltransferase E	TGM3_HUMAN	C, rpAD	Cytoplasm	Keratinization
64	Tripeptidyl-peptidase 1	TPP1_HUMAN	AD	Lysosome	Proteolysis

65	Tubulin polymerization-promoting protein	TPPP_HUMAN	CJD	Cytoskeleton, Nucleus	Microtubule polymerization
66	Transthyretin	TTHY_HUMAN	C	Secreted	Extracellular matrix organization
67	Ubiquitin carboxyl-terminal hydrolase isozyme L1	UCHL1_HUMAN	AD	Cytoplasm	Ubiquitination
68	V-type proton ATPase subunit H	VATH_HUMAN	C, AD	Cytosol and plasma membrane	Ion transport
